# Classification of protein–protein association rates based on biophysical informatics

**DOI:** 10.1186/s12859-021-04323-0

**Published:** 2021-08-17

**Authors:** Kalyani Dhusia, Yinghao Wu

**Affiliations:** Department of Systems and Computational Biology, Albert Einstein College of Medicine, 1300 Morris Park Avenue, Bronx, NY 10461 USA

**Keywords:** Protein–protein association, Kinetic Monte-Carlo simulation, Neural network model

## Abstract

**Background:**

Proteins form various complexes to carry out their versatile functions in cells. The dynamic properties of protein complex formation are mainly characterized by the association rates which measures how fast these complexes can be formed. It was experimentally observed that the association rates span an extremely wide range with over ten orders of magnitudes. Identification of association rates within this spectrum for specific protein complexes is therefore essential for us to understand their functional roles.

**Results:**

To tackle this problem, we integrate physics-based coarse-grained simulations into a neural-network-based classification model to estimate the range of association rates for protein complexes in a large-scale benchmark set. The cross-validation results show that, when an optimal threshold was selected, we can reach the best performance with specificity, precision, sensitivity and overall accuracy all higher than 70%. The quality of our cross-validation data has also been testified by further statistical analysis. Additionally, given an independent testing set, we can successfully predict the group of association rates for eight protein complexes out of ten. Finally, the analysis of failed cases suggests the future implementation of conformational dynamics into simulation can further improve model.

**Conclusions:**

In summary, this study demonstrated that a new modeling framework that combines biophysical simulations with bioinformatics approaches is able to identify protein–protein interactions with low association rates from those with higher association rates. This method thereby can serve as a useful addition to a collection of existing experimental approaches that measure biomolecular recognition.

**Supplementary Information:**

The online version contains supplementary material available at 10.1186/s12859-021-04323-0.

## Background

The formation of various protein complexes is building blocks for nearly all physiological processes [1–5]. The association rate ($k_{ass}$) which measures how fast proteins form a complex is of fundamental importance to characterize its function [6]. In a crowded environment of cells, different proteins might compete for their binding partners. The dynamics of a biological system is usually not under thermodynamic, but under kinetic control [7], in which the range of association rates for proteins in the system plays a critical role. For instance, the binding kinetics between ligands and membrane receptors control the speed of signal transduction after they are exposed to extracellular stimulations. The observed values of association rate constants span an extremely wide range with over ten orders of magnitudes [8–14]. Identification of association rates for binding between different proteins within this spectrum is essential for us to understand their functional roles in signal transduction, transcriptional regulation, and many other cellular activities [15–18]. For instance, natural-killer (NK) cell receptor NKG2D (natural-killer group 2, member D) recognizes both cellular and viral ligands with the same binding interface, indicating that these ligands have to compete with each other for receptor binding when they coexist in the system [19]. The difference in association rates of receptor binding between cellular and viral ligands directly regulates the NK cytolytic activity. Another example is the difference in association of binding between receptor activator of nuclear factor-κB ligand (RANKL), and its receptor, receptor activator of nuclear factor-κB (RANK), from the binding to its competitor osteoprotegerin (OPG) [20]. Tthe difference in association rates of RANK binding between RANKL and OPG determines the ultimate rate of bone resorption. These examples highlight the significance to quantitatively estimate protein–protein association rates.

Fortunately, nowadays a large variety of mature experimental techniques, such as surface plasma resonance (SPR) [21] and spectroscopic inhibition assay (IASP) [22], are available to measure rate constants of protein–protein interactions. Moreover, the information of many experimentally measured binding constants has been collected in different publically accessible databases. For an example, SKEMPI contains data on thermodynamic parameters and kinetic rate of more than one hundred protein–protein interactions and thousands of relevant mutations [23]. The structure of these protein complexes has also been solved and is available in the Protein Databank. These experimental data facilitate the development of computational approaches to model and predict protein–protein association, which are much less time-consuming and labor-intensive comparing with traditional experimental techniques. One type of these computational approaches, including molecular dynamic (MD) [24, 25] or Brownian dynamic (BD) simulations, is based on physics-based principles to reproduce the association processes between proteins [26–48]. These all atom-based methods, however, are computationally expensive. Different levels of coarse-grained (CG) models therefore have been developed to simplify protein structures [49]. These models have been applied to study protein folding and aggregation [50, 51]. In contrast, the other type of computational approaches utilized artificial-intelligence-based algorithms to predict association rate constants based on the chemical or structural features embedded in the binding interfaces of protein complexes [52, 53]. These prediction methods, however, are lack of the information that describes detailed mechanisms along the pathways of association.

**Fig. 1:**
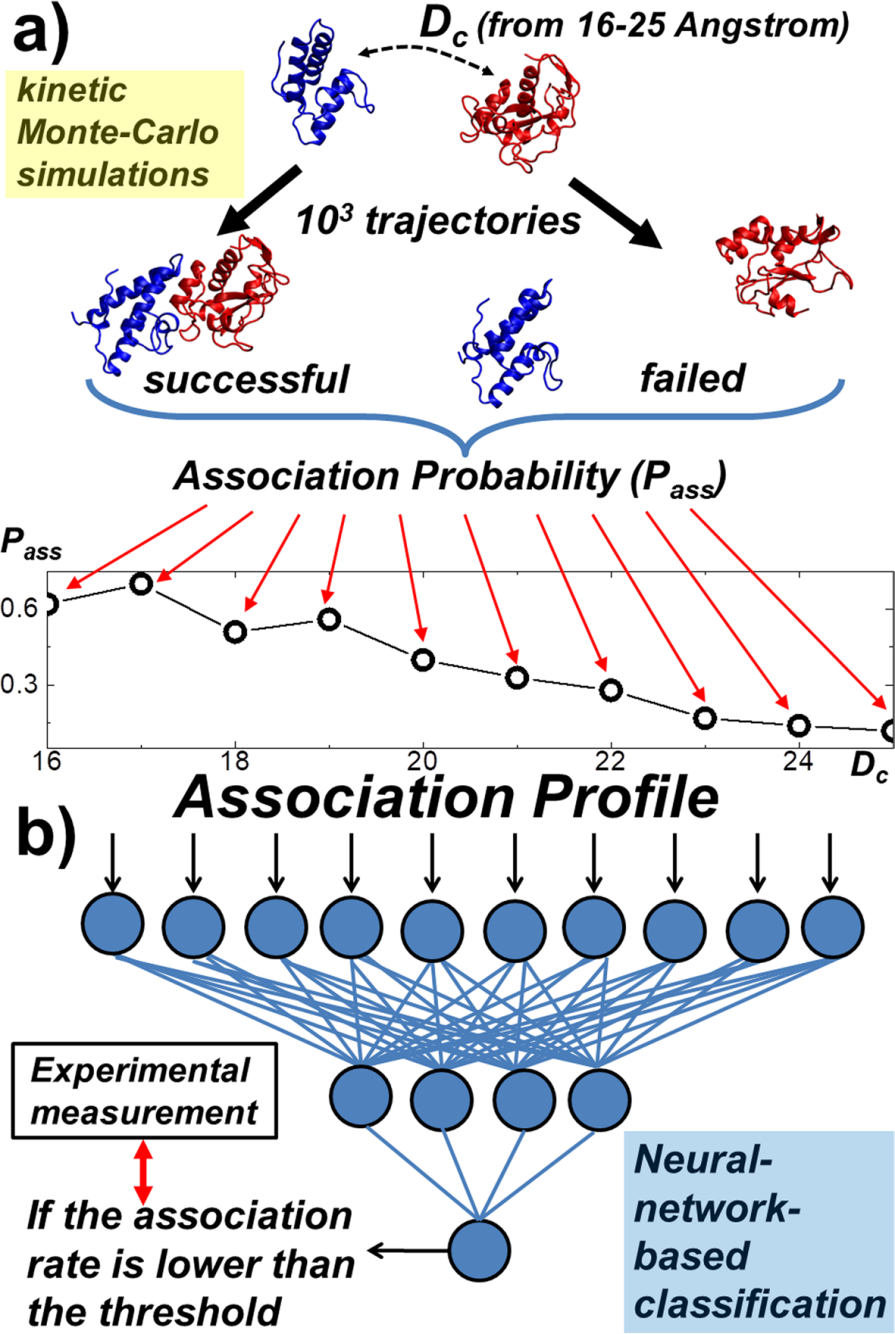
The flowchart of the overall computational procedure. A coarse-grained Monte-Carlo simulation was used to generate a large number of protein–protein association trajectories (**a**). Based on counting how many encounter complexes were formed among all the trajectories, the association probability can be derived. Simulations are further carried out under different values of distance cutoff, so that a profile of association probabilities was generated for each protein complex in the benchmark set. Using these association profiles as input, a back propagation neural network algorithm was then applied to classify whether the association rate of a protein complex is higher than a predefined threshold (**b**)

Different from either type of above-mentioned computational methods, here we present a platform that combines both biophysics-based simulations with bioinformatics-based prediction to classify rates of protein–protein association presented in the SKEMPI database. A previously developed coarse-grained Monte-Carlo simulation (Fig. 1a) was used to generate a large number of protein–protein association trajectories [54]. In this method, each residue is simplified by its Cα atom plus a representative center on the side-chain. Random diffusions are carried out to a pair of initially separate interacting proteins, under the guidance of a simple physics-based force field. The probabilities of association can thus be calculated by counting the frequency of forming the encounter complex between these two proteins among a large number of simulation trajectories. The back propagation neural network algorithm (Fig. 1b) was then applied to classify the probabilities of association that were derived from these coarse-grained simulations. Based on the cross-validation results, we show that this method can achieve the best performance with specificity, precision, sensitivity and overall accuracy all higher than 70%. Given an independent testing set, we can further successfully predict the group of association rates for eight protein complexes out of ten. Finally, the analysis of failed cases suggests the future implementation of conformational dynamics into simulation can further improve model. In summary, this study indicates that a new modeling framework using tools of biophysical informatics is able to identify protein–protein interactions with low association rates from those with higher association rates. This method serves as a useful addition to a collection of existing experimental approaches that measure biomolecular recognition.

## Results

We first applied kinetic Monte-Carlo (kMC) method to estimate the probability of association under different initial separation for each protein complex in a large-scale benchmark set. The detailed information about the construction of this benchmark and the algorithm of the kMC simulation can be found in the “Methods”. Specifically, for each of the 102 protein complex systems, a large number of simulation trajectories were generated. As the initial conformation of each trajectory, the residue-based coarse-grained models of two binding partners in a complex were individually placed with a random position relative to each other, in which the distance between their binding interfaces is fallen within a cutoff value *d*_*c*_. We systematically tested ten different values of distance cutoff from 16 to 25 Å. For each value of *d*_*c*_, 10^3^ simulation trajectories were carried out. The initial conformations in these trajectories are different from each other. After the initial conformation, as described in the “Methods”, diffusions of each binding are guided by the intermolecular energies which contain both hydrophobic effect and electrostatic interactions. At the end of all these trajectories, two binding partners either form an encounter complex through the pre-defined association criteria, or diffuse further apart from each other. Based on the simulation results collected from all the trajectories, the association probability under a given a specific value of *d*_*c*_ can be calculated for each complex in the benchmark set.

**Fig. 2:**
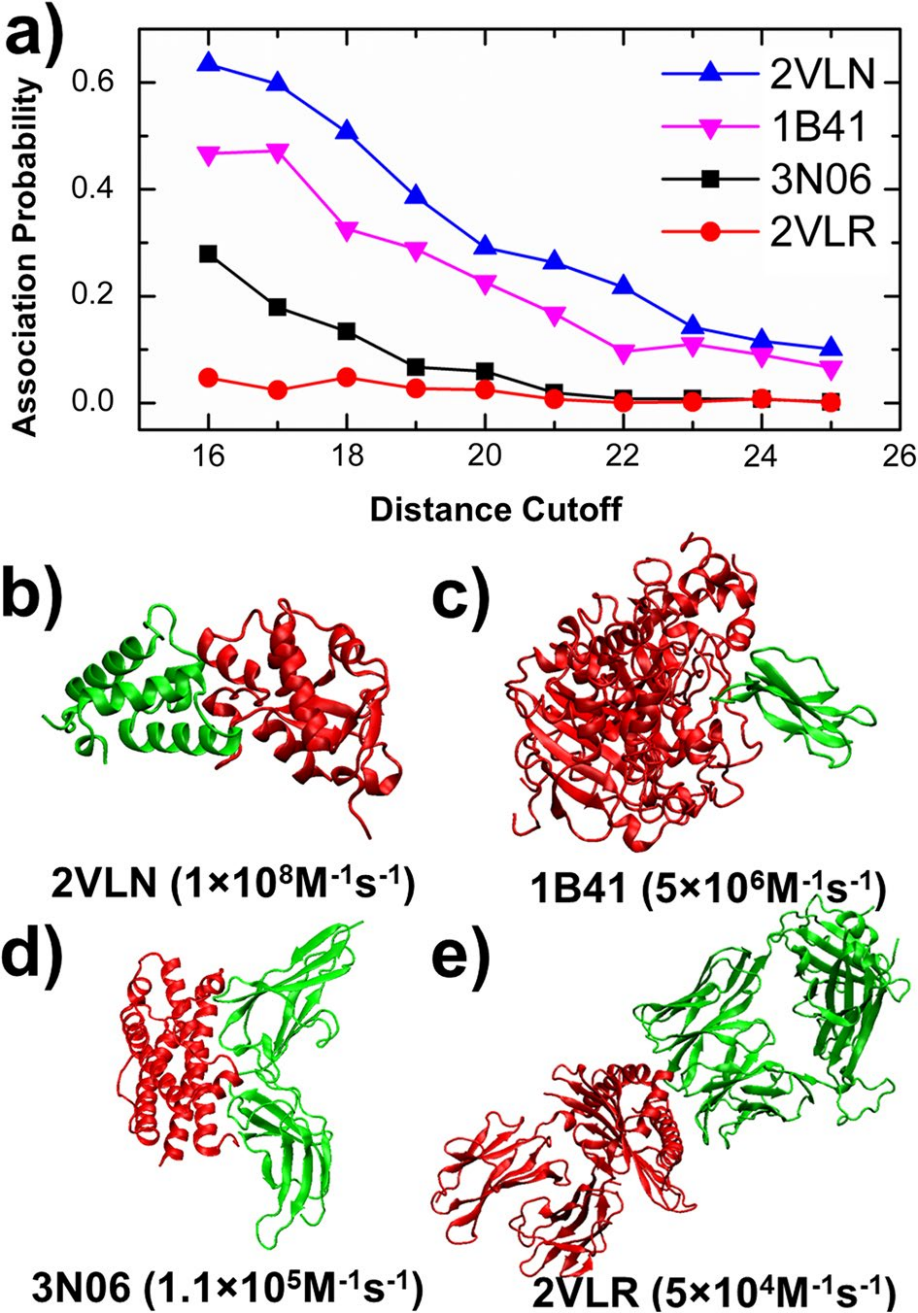
The outputs from the kinetic Monte-Carlo simulations. For each complex, a large number of simulation trajectories were generated under ten different values of distance cutoff. The plot (**a**) shows the relations between distance cutoff and association probabilities for four selected systems. They are E9 DNase domain of Colicin Endonucleases in complex with immunity protein Im9 (**b**); human acetylcholinesterase in complex with the snake-venom toxin fasciculin-II (**c**); human prolactin receptor antagonist H27A in complex with the extracellular domain of the human prolactin receptor (**d**); and the HLA class I histocompatibility antigen in complex with β-2 microglobulin (**e**). Their PDB identities and experimentally measured association rates are listed in the bottom

Among all the 102 protein complexes, we successfully generated all 10^3^ simulation trajectories for 96 complexes under all 10 distance cutoff values, and failed to complete all the simulation runs for 6 complexes. As a result, these 6 systems were not considered in the following study. For the remaining 96 systems, the relation between *d*_*c*_ and its corresponding association probability was used as the basis for further study, in which neural network model will be used to differentiate association rates between various protein complexes. The relations between different values of *d*_*c*_ and association probabilities were selected and plotted in Fig. 2a for four representative systems. They are: E9 DNase domain of Colicin Endonucleases in complex with immunity protein Im9 (PDB 2VLN); human acetylcholinesterase in complex with the snake-venom toxin fasciculin-II (PDB 1B41); human prolactin receptor antagonist H27A in complex with the extracellular domain of the human prolactin receptor (PDB 3N06); and the HLA class I histocompatibility antigen in complex with β-2 microglobulin (PDB 2VLR). The corresponding structures of these complexes are shown in Fig. 2b–e. Two binding partners in the complexes are indexed in red and green, respectively. Their PDB identities and experimentally measured association rates are also listed in the bottom. Figure 2a shows that probabilities of association drop for all the four systems when the distance cutoff increases, suggesting that complexes are more difficult to form if the initial separation of two binding partners are farther away from each other in the beginning. Moreover, the figure shows that the overall association probabilities for the complexes with larger values of experimental association rates are higher than the complexes with smaller values of experimental association rates. For instance, the overall association probabilities of complex 2VLN are higher than the other three complexes in Fig. 2a, while the association probabilities of complex 2VLR is the lowest. Correspondingly, the experimental association rate of 2VLN is 1 × 10^8^ M^−1^ s^−1^, the highest among these four systems. Similarly, the experimental association rate of 2VLR is the slowest (5 × 10^4^ M^−1^ s^−1^).

**Fig. 3:**
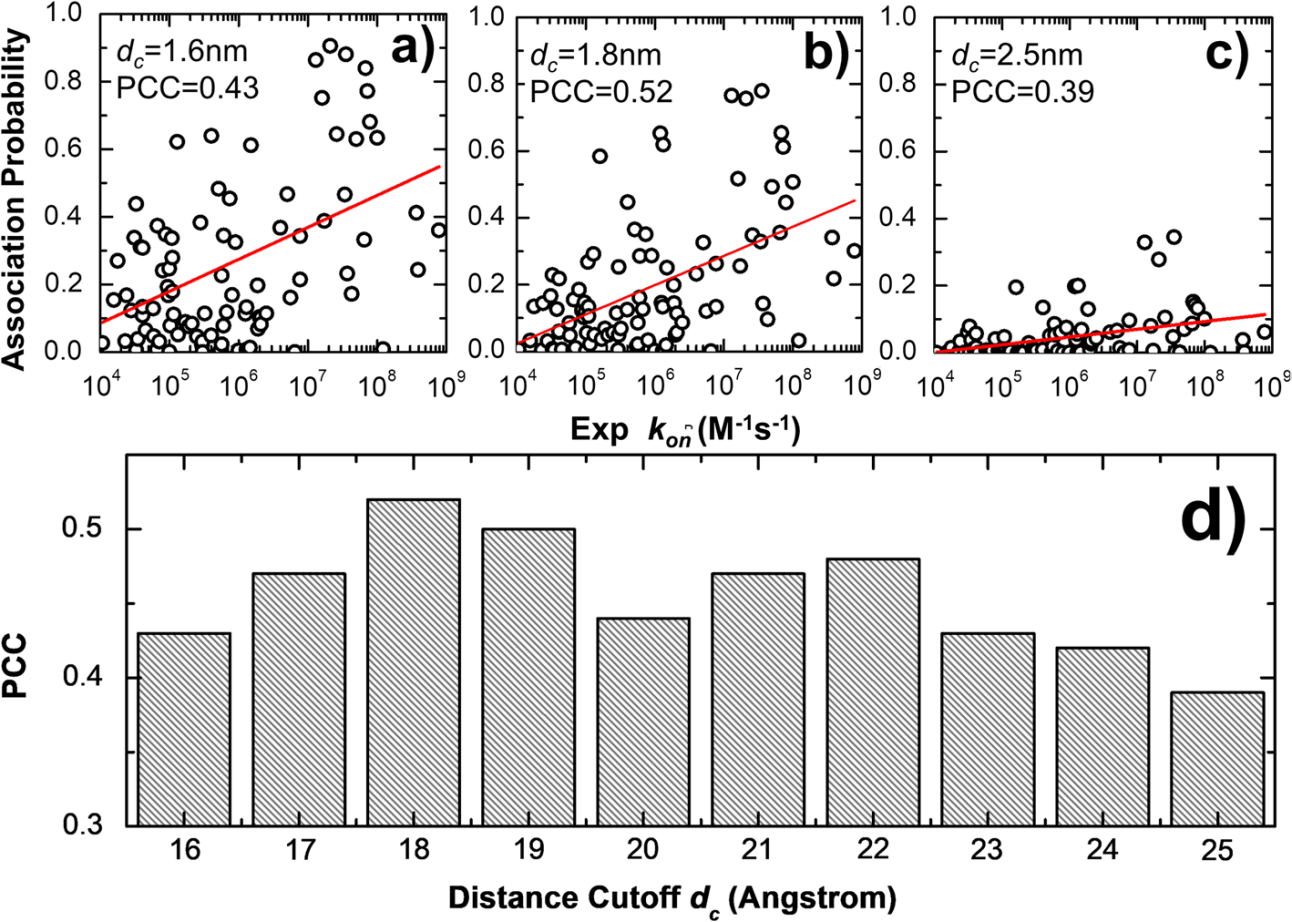
The correlations between simulated association probabilities and their corresponding experimental measurements. In specific, the correlation between simulated association probabilities and experimental association rates for all 96 protein complexes given a distance cutoff of 16 Å, 18 Å, and 25 Å are plotted in (**a**), (**b**), and (**c**), respectively. To compare simulated association probabilities with experimental association rates on a more quantitative level, we further calculated the Pearson correlation coefficient (PCC) between these two data sets as a function of distance cutoff (**d**)

To generalize our study, we further tested the correlation between simulated association probabilities and their experimental measurements for all 96 protein complexes in the benchmark under different distance cutoff values (Fig. 3). All association probabilities of 96 protein complexes with a distance cutoff of 16 Å are plotted as circles in Fig. 3a. The y axis in the figure indicates the simulated association probabilities and x axis is the experimental data with the scale in common logarithm. Similarly, the association probabilities with a distance cutoff of 18 Å are plotted in Fig. 3b, while the association probabilities with a distance cutoff of 25 Å are plotted in Fig. 3c. When the distance cutoff increases, we found that the association probabilities drop for most protein complexes in the benchmark, which is consistent with the results reflected from Fig. 2a. Moreover, the Pearson's correlation coefficient (PCC) for all protein complexes between their simulated association probabilities and experimentally derived association rates were calculated under different distance cutoff. These PCC values are plotted as histogram in Fig. 3d. Positive PCC values were observed under all distance cutoff. The PCC equals 0.43 when distance cutoff is 16 Å (Fig. 3a). It increases to the maximal value of 0.52 when distance cutoff equals 18 Å (Fig. 3b). Afterwards, the level of PCC becomes lower and it equals 0.39 when distance cutoff finally reaches 25 Å (Fig. 3c), These positive correlations suggest that the Monte-Carlo simulations on average can distinguish fast from slow kinetics within a wide range of protein–protein associations.

On the other hand, we also noticed that these positive correlations are only moderate. More specifically, there are still a large number of outliers with high simulated association probabilities but low experimentally measured association rates, or with low simulated association probabilities but high experimentally measured association rates. However, it is worth mentioning that these association probabilities were derived from different distance cutoff. The association of a protein complex is a complicated process, and the pathways in the association of different protein complexes are case-dependent. It is possible that a protein complex with low experimental association rates but has high simulated association probabilities at certain distance cutoff, or vice versa. Instead of focusing on the association probabilities under single distance cutoff, it would be more informative to compare association probabilities from different distance cutoff values. As shown in Fig. 1, the association profile for a protein complex delineates the variations of association probabilities under different distance cutoff. We hypothesize that features of association pathways for different protein complexes can be reflected by the patterns of these association profiles. Moreover, these high-dimensional patterns can be identified from each other by methods that are beyond the physics-based simulations. As a result, a neural-network-based classification model was further integrated into our coarse-grained simulation results to give a systematic estimation on how associations of different protein complexes within this wide range of rate constants can be identified with each other.

**Fig. 4:**
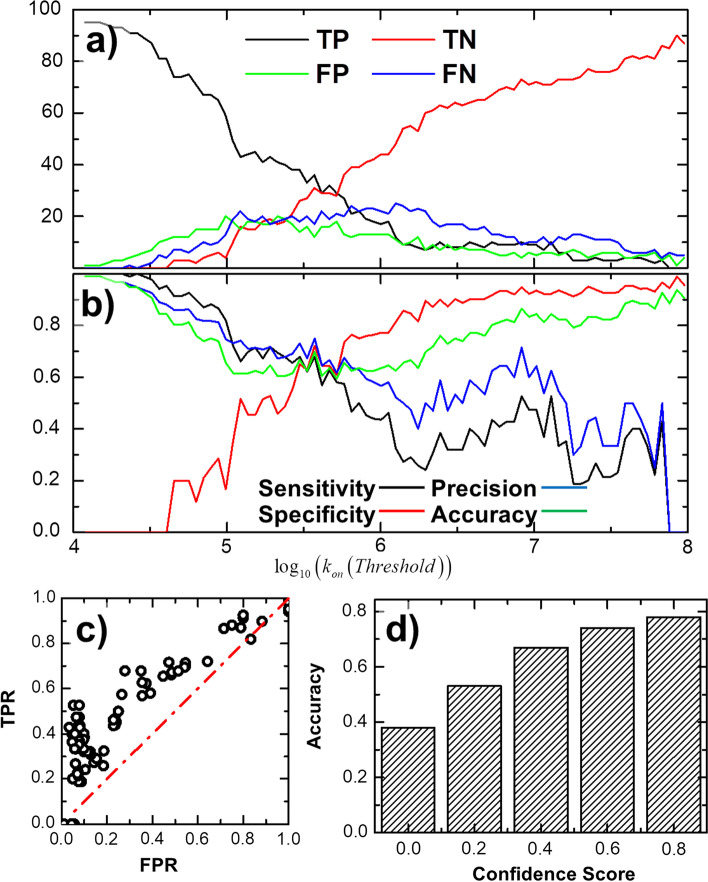
The overall performance of neural-network-based classification. We plotted the true positive, true negative, false positive and false negative (**a**), as well as the specificity, sensitivity, precision and overall accuracy (**b**) as a function of classification threshold. We also compared the true positive rate with the false positive rate from the classification results under different threshold values. The results correspond to a receiver operating characteristic (ROC) curve, as shown in (**c**). Finally, we found that the classification accuracy has a positive correlation with the confidence score offered by the neural network model (**d**)

In detail, a feedforward back-propagation algorithm was utilized to estimate whether the association rate of a protein complex is higher or lower than a predefined threshold. In specific, the input of the classification model is in ten dimensions, which are the association profile of a protein complex that was generated from kMC simulation with the distance cutoff values between 16 and 25 Å (Fig. 1a), while the binary output of the model is simply the information about if the association rate of the complex is higher than the threshold or not. As described in the “Methods”, a leave-one-out cross-validation strategy has been applied to classify all protein complexes in the benchmark set. In order to calibrate the performance of the cross-validation, we respectively counted the numbers of true positive (TP), true negative (TN), false positive (FP) and false negative (FN) from the classification results. A TP or TN is recognized if we correctly classified a protein complex which experimental association rate is higher or lower than the threshold, respectively. Relatively, a FP or FN is recognized if a protein complex is classified higher or lower than the threshold but its actual experimental association rate is on the opposite side of the threshold. Our results are shown in Fig. 4a as a function of the threshold values. The figure indicates that while the value of threshold becomes larger, the number of TP decreases and the number of TN increases monotonously. On the other hand, the number of FP and FN increase at the beginning but decrease later.

The values of sensitivity (TP/(TP + FN)), specificity (TN/(TN + FP)), precision (TP/(TP + FP)), and overall accuracy ((TP + TN)/(TP + TN + FP + FN)) were further calculated [55]. Figure 4b plots the calculated results under different threshold values. The x-axis in the figure is the threshold of association rate under logarithm scale, while the sensitivity, specificity, precision and overall accuracy are shown in black, red, blue and green along the y-axis, respectively. Figure 4b indicates that the sensitivity and precision decrease to 0, while the specificity increases to the maximal level along with the raise of threshold from small to large values. This is because under larger values of threshold, fewer protein complexes are classified as TP (the experimental association rates higher than the threshold). Based on the definitions of sensitivity and precision, in which TP is presented as numerator, their values are negatively correlated with the increase of threshold. On the other hand, under larger values of threshold, more protein complexes are classified as TN (the experimental association rates lower than the threshold). Based on the definition of specificity, in which TN is presented as numerator, its values are positively correlated with the increase of threshold.

Figure 4b shows that, when the threshold equals 4 × 10^5^ M^−1^ s^−1^, corresponding to the logarithm value of 5.6 in the figure, the system achieves the optimal performance in which specificity, precision, sensitivity and accuracy all higher than 70%. Among all the 96 protein complexes in the benchmark set, there are 43 complexes which experimental association rates are lower than this optimal threshold and 53 complexes which experimental association rates are higher than the cutoff. Therefore, optimal performance was obtained when the testing set fall into two classes which sizes are relatively close to each other. Moreover, the neural network model can provide a confidence score (between 0 and 1) for each classification. Therefore, under the threshold of 4 × 10^5^ M^−1^ s^−1^, we further broke down the accuracy of classification into different intervals of confidence score. As shown in Fig. 4d, the classification accuracy has a positive correlation with the model confidence score. For those protein complexes that were classified by the model with high confidence score (higher than 0.8), the best accuracy of 77% can be achieved. The individual cross-validation results based on the optimal threshold are summarized by Table S2 in the Additional file 1 for all protein complexes in the benchmark, and can also be found at https://github.com/wulab-github/KonPred.

A statistical analysis has further been carried out to test the classification results. In detail, after cross-validation was performed to all protein complexes in the benchmark for a given threshold, we also investigated the correlation between true positive rate (TPR) and false positive rate (FPR) from the classification results. The TPR is equivalent to sensitivity, based on the definition. The FPR, on the other hand, is defined as the ratio between the total number of FP versus the summation of FP and TN. Practically, both TPR and FPR are covariant with the choice of threshold. We thus changed the value of the threshold gradually from 1 × 10^4^ to 1 × 10^9^ M^−1^ s^−1^ and monitored the correlated changes between TPR and FPR, leading into a collection of points as shown in Fig. 4c. Statistically, these points correspond to a receiver operating characteristic (ROC) curve [56, 57], and are compared with the red diagonal which is known as the line of no-discrimination indicating that the test is completely based on random guess. Figure 4c shows that the TPRs under all different values of threshold are consistently higher than the FPRs. For instance, we obtained a TPR of 0.7 when FPR equals 0.3. Therefore, the ROC curve represents the good quality of our classification data.

In order to assess how significantly our obtained classification performance can be distinguished from random estimation, we carried out predictions with two different models. In one model, predictions were made by our neural-network-based classification method with the optimal threshold. In the control model, predictions were made purely by random guessing. Each protein complex was randomly assigned either higher or lower than the optimal threshold with equal probabilities. Predictions were carried out for all 96 protein complexes in the benchmark. This process was repeated 100 times for both models. After the predictions, the values of sensitivity, specificity, precision and accuracy were calculated, which distributions were plotted and compared in Additional file 1: Figure S1. The black histograms in the figure are the distribution from the predictions based on our neural-network model, while the red histograms are the distribution from random estimation. The average values and corresponding standard deviations can be found in Additional file 1: Table S1. Student’s t-tests were further performed to testify the statistical significance in the difference between the prediction results of two models. The null hypothesis that no significant difference exists between the results from these two models was tested at a 95% confidence interval. Consequently, the derived t-scores equal 21.79, 25.15, 34.71 and 35.88, in the comparison of sensitivity, specificity, precision and accuracy, respectively. The corresponding *P* values for all these tests are less than 0.0001. Therefore, the small *P* value for the t-test suggests that we can reject the null hypothesis and accept the alternative hypothesis, i.e., the differences between the outputs generated from our neural-network-based model and the outputs generated by random guessing are significant.

In summary, the statistical analysis on the cross-validation results demonstrated that we are able to identify protein complexes with high association rates from others with low association rates using a reliable and accurate model that combines biophysics-based simulations and machine-learning-based bioinformatics algorithm.

**Fig. 5:**
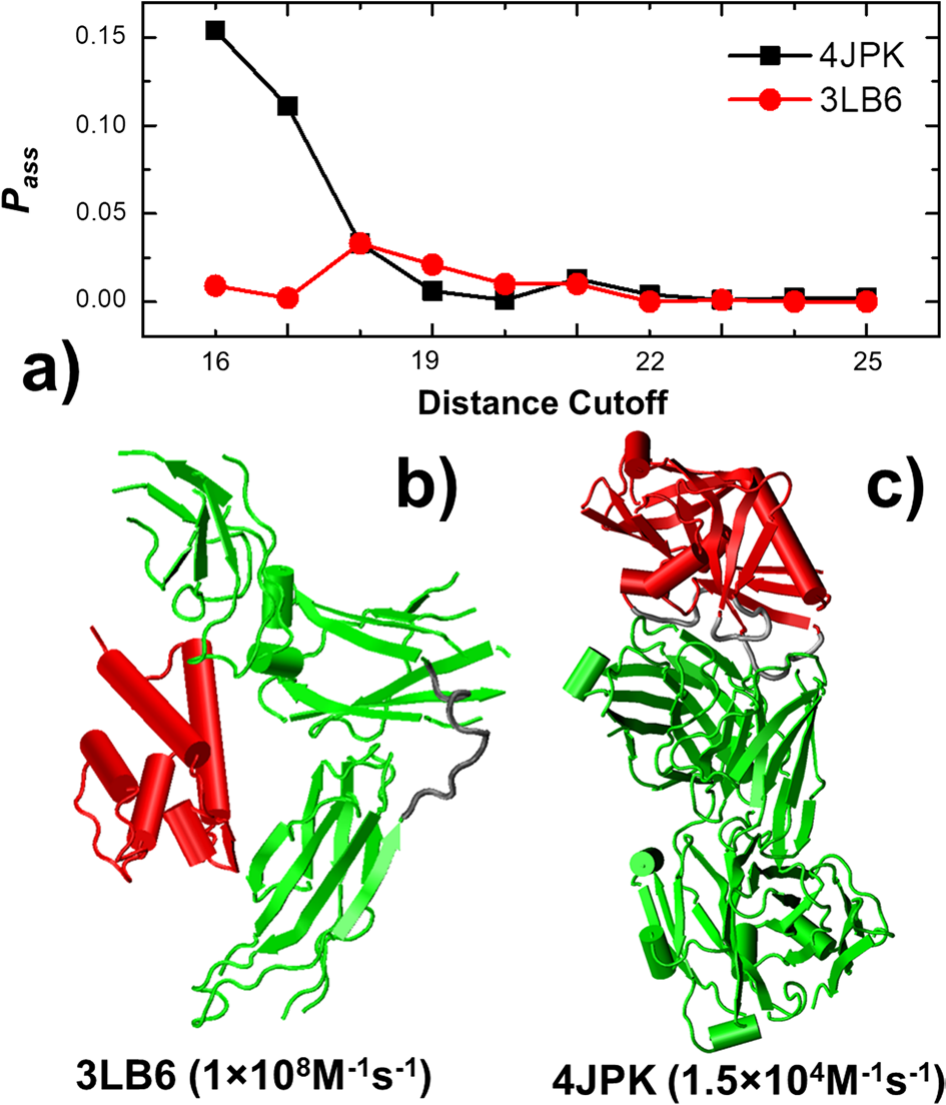
The individual cases in which our method failed to generate the correct output. The plot (**a**) shows the relations between distance cutoff and association probabilities for these two systems. They are: cytokine Interleukin-13 (IL-13) in complex with its receptor IL-13 Receptor α2 (IL-13Rα2) (**b**), and an engineered outer domain of envelope glycoprotein GP120 from human immunodeficiency virus 1 in complex with a VRC01-class broadly neutralizing antibodies (**c**). The corresponding structures of two binding partners in the complexes are indexed in red and green, while the flexible regions that undergo large conformational fluctuations are highlighted in gray. Their PDB identities and experimentally measured association rates are also listed in the bottom

Although our test proved that the association profiles for most protein complexes can be successfully recognized, there are still possibilities that complexes were classified into wrong groups. Figure 5 shows two individual cases in which our method failed to generate the correct output. One is cytokine Interleukin-13 (IL-13) in complex with its receptor IL-13 Receptor α2 (IL-13Rα2) (PDB 3LB6), while the other is an engineered outer domain of envelope glycoprotein GP120 from human immunodeficiency virus 1 (HIV-1) in complex with a VRC01-class broadly neutralizing antibodies (bNAbs) (PDB 4JPK). The association profiles of these two protein complexes generated from kMC simulations are plotted in Fig. 5a. The figure shows that the overall simulated association probabilities of 4JPK (black squares) are much higher than 3LB6 (red dots), although the experimentally measured association rate of 4JPK (1.5 × 10^4^ M^−1^ s^−1^) is much slower than 3LB6 (1 × 10^8^ M^−1^ s^−1^). As a result, neither 4JPK nor 3LB6 has been classified into the correct group by neural network model. The association rate of 4JPK was identified to be above the threshold 4 × 10^5^ M^−1^ s^−1^, while the association rate of 3LB6 was identified to be below the threshold. In order to explore the reason why our simulations generated the results that are opposite to the experimental measurements, we plotted the structures of these two complexes in Fig. 5b, c. Figure 5b shows the complex 3LB6, in which the cytokine is shown in red and the receptor is shown in green. IL-13 is important for the development of T helper cell type 2 (Th2) responses and plays a critical role in asthma and allergy. Its interaction with the receptor IL-13Rα2 has high association rate and binding affinity. The structure of the cytokine-receptor complex in Fig. 5b shows that the receptor has three fibronectin domains connected by domain linkers which are highlighted in grey in the figure. The binding interfaces in the receptor to the cytokine are equally distributed on all its three domains. It has been suggested that associations of multi-domain proteins with flexible linkers are completed through a multistep “dock-and-coalesce” mechanism [4, 58, 59]. Association can be greatly accelerated by this mechanism, in which the conformational flexibility of proteins plays a critical role. The intramolecular flexibility is neglected in our kMC simulations. This could be the reason that our estimated association rated is much lower than the real value. On the contrary, the recognition of GP120 outer domain (red in Fig. 5c) by the antibody (green in Fig. 5c) is close regulated by the hypervariable loops at the binding interface of the antibody [60], as highlighted by grey in the figure. The local conformational dynamics of the flexible loops at the binding interface can impede its association with the viral protein. Similarly, because the intramolecular flexibility is neglected in our kMC simulations, which could result in the result that our estimated association rated is much higher than the real value. As a result, our test highlighted the importance of protein local dynamics and global conformational changes in regulating the protein–protein association. Our method can potentially be improved in the future by the implementation of conformational dynamics into the kMC simulation.

To further test the stability of our classification model and rule out the possibility of over-fitting, an additional test set consisting of 10 protein complexes was independently collected. The detailed information of these complexes can be found in the Table 1. Under each of the 10 different distance cutoff values from 16 to 25 Å, multiple trajectories (10^3^) were carried out with different random initial configurations based on the reported ionic strength. Their corresponding association profiles were then calculated from the simulations. Using these profiles as inputs, the neural network model was further utilized to estimate whether the association rates of these 10 protein complexes are higher or lower than the optimized threshold (4 × 10^5^ M^−1^ s^−1^). During the classification, the association profiles of all the 96 protein complexes in the benchmark and their corresponding experimental association rates were treated as the training set, while each of the 10 protein complexes was fed into the neural network for testing. The prediction was further compared with the real experimental data. As a result, we found that we can correctly predict if the association rates are faster or slower than the threshold for eight protein complexes out of ten, consistent with the cross-validation results.

**Table 1:** The detailed information of an independent test set

PDB ID	Chain 1	Chain 2	Ionic strength (M)	Correctly classified?	*k*_*on*_ (exp.) (M^−1^ s^−1^)
1KAC	A	B	0.16	Y	7.30E+04
1SBB	A	B	0.16	Y	1.00E+05
2I25	N	L	0.16	N	9.00E+04
2J0T	A	D	0.23	Y	2.40E+04
2A22	A	B	0.025	Y	1.50E+05
1EWY	A	C	0.31	Y	4.00E+07
1SGN	E	I	0.26	N	1.20E+06
1TLU	A	B	0.01	Y	5.60E+06
1UDI	E	I	0.08	Y	1.50E+08
7CEI	A	B	0.25	Y	7.60E+08

**Fig. 6:**
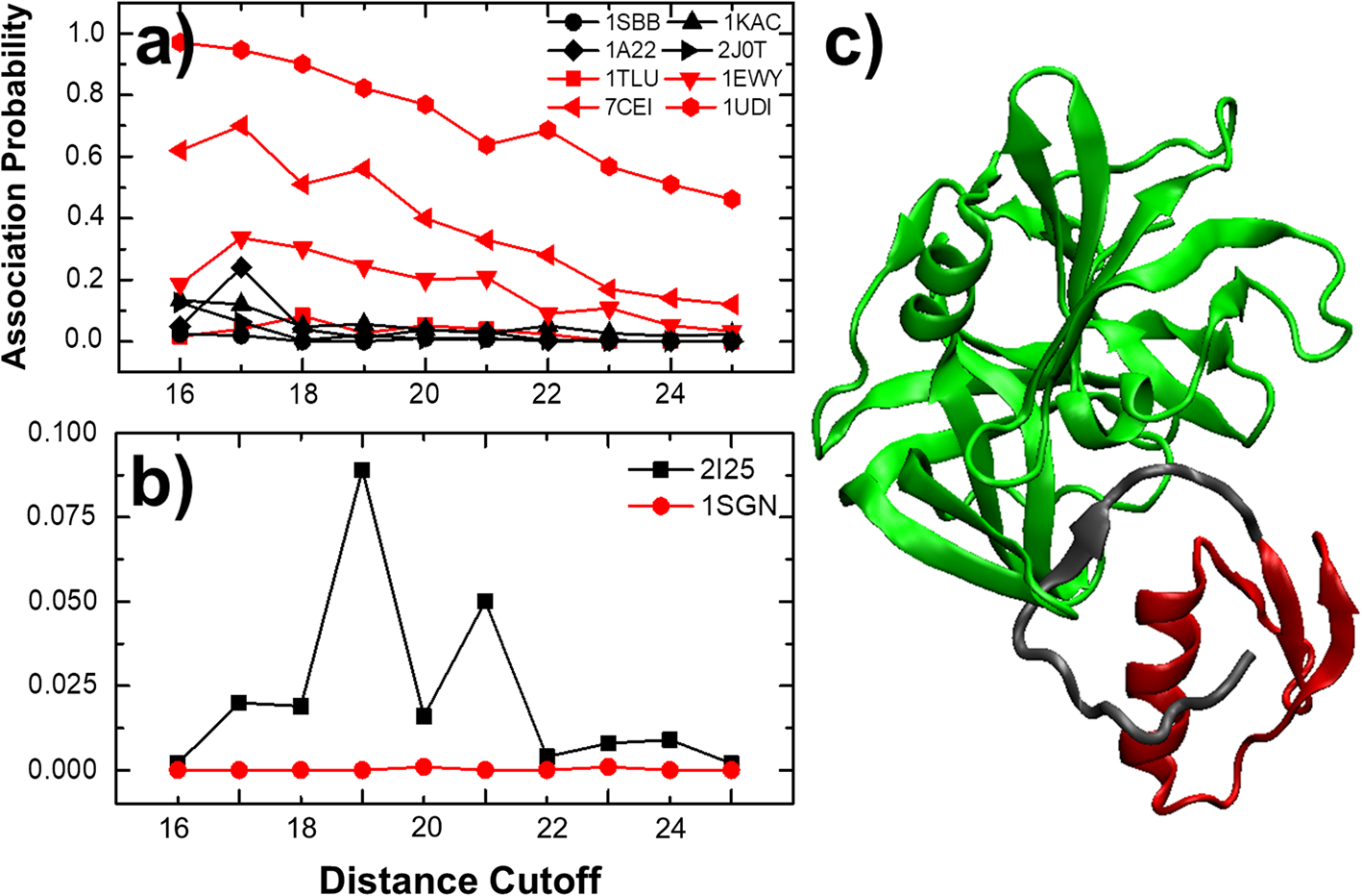
The classification results of an independent test set. The association profiles of 8 successfully predicted cases are plotted in (**a**), while the rest 2 incorrectly predicted cases (2I25 and 1SGN) are plotted in (**b**). Moreover, the structure of a complex (1SGN) that we failed in classification is shown in (**c**)

Our prediction results are summarized in Fig. 6. The association profiles of 8 successfully predicted cases are plotted in Fig. 6a, while the rest 2 incorrectly predicted cases (2I25 and 1SGN) are plotted in Fig. 6b. The profiles with experimental association rates higher than the threshold are shown in red, while the profiles with experimental rates lower than the threshold are shown in black. The association profiles of complexes 1EWY, 1UDI and 7CEI are the highest among all the ten and thus can be easily identified. The association profile of complex 1TLU (red squares in Fig. 6a), on the other hand, is mixed with the profiles of the other class, but still was successfully recognized. Finally, the association profile of complex 1SGN (red circles in Fig. 6b) is lower than all the other complexes, although its experimentally measured rate constant is 1.2 × 10^6^ M^−1^ s^−1^. Not surprisingly, it has been incorrectly assigned to the class with association rate below the threshold. 1SGN is a complex formed between protein Ovomucoid and Streptogrisin B. A closer structural inspection indicates that the interaction of the complex is formed through an inter-molecular β-sheet (Fig. 6c). Without forming complex, the β-strand from the protein Ovomucoid (red in Fig. 6c) might exist as an intrinsic disordered region (highlighted in gray). This conformational transition upon association cannot be considered in our simulations, and the association rate of the complex thus was underestimated. Taken together, our independent test demonstrated the stability of our computational method in protein–protein association rate classification and there is no over-fitting in the leave-one-out cross-validation procedure.

## Discussion

A periodic boundary condition was applied in our previous simulation study. As a result, the association rates were directly derived based on the predefined volume of simulation box. However, significant overestimation was observed for a group of protein complexes, comparing the calculated association rates with their experimental values. We assume that the effect of long-rang interactions between proteins on their association might not be appropriately captured by fixing the size of simulation box. Therefore, a new strategy was adopted in current study. Instead of using the periodic boundary condition, a pair of interacting proteins can freely diffuse from different distance cutoffs, and the probabilities of forming an encounter complex were then separately calculated. These high-dimensional profiles of association probability for different protein complexes were then characterized by artificial intelligence so that their association rates can be classified based on the experimental observations. As we mentioned in the introduction, these experimentally observed association rates form an extremely wide spectrum. If we can predict the range of association rates for a protein–protein interaction, it would help us understand its biological function in the cellular context.

There are still some limitations in the current model which can be improved in the future. First, when we generated initial conformations, we separated two binding partners of a protein complex and calculated the distance between residues in their native binding interfaces. Similarly, when we judged if encounter complexes have been formed or not, we checked if the native-like inter-molecular interactions have been restored. In another word, the basis of our method is that we have already known the structure of a protein complex which association rate is unknown and needs to be predicted. In order to apply our method to protein complexes with unknown structures, computational modeling methods such as TACOS [61] can be integrated into our prediction prior to our Monte-Carlo simulations to construct the initial structural models of query protein complexes. Second, when we set up initial configurations, the distance between binding interfaces is used as the only criterion. Other characteristics of protein complexes such their sizes or the chemical properties on the binding interfaces were neglected in current study. How these features embedded in the ensemble of initial conformations and how they specifically regulate the association of different protein complexes will be considered in the future.

Finally, in current study the results from the Monte-Carlo simulations are used as the only inputs of the neural network classification. Features like the structural characteristics of the protein complexes at their binding interfaces including size, charge distribution, hydrophobicity, or intrinsic flexibility can also be added to enrich the inputs, which could allow the neural network to extract some additional patterns. As a preliminary test, the information about the number of intermolecular contacts between residues at native binding interface was used as an independent dimension to train the neural network, together with the original inputs from Monte-Carlo simulations. The testing results show that it did not improve the prediction (Additional file 1: Figure S6a), probably due to the low correlation between the numbers of native contacts and the experimental association rates (Additional file 1: Figure S6b). Therefore, input features need to be carefully selected in the future to ensure more meaningful outputs from the neural network model.

On the other side of the model, only the position with respect to a threshold value can be predicted from current study. The method will definitely be much more useful if a richer output, such as more specific ranges or values of association rates, can be given. In order to reach this goal, more sophisticated algorithms of artificial intelligent such as support vector regression will be implemented into our prediction framework [62]. However, we need to point out that estimating whether the rates of association are faster or slower than a threshold alone can still be important to understand the molecular mechanism of protein–protein interactions. Previous works showed that the association rate constant of forming transient complexes purely via unbiased diffusions is on the level of 10^5^ M^−1^ s^−1^ [41, 63, 64], corresponding to the optimal threshold in current study. The real values of association rate higher than this “basal” rate constant are originated from the intermolecular interactions in a protein complex such as the long-range electrostatic attraction. As a result, the association rates calculated from computational models that can differ from several orders of magnitude, such as the method developed in this study, can thus help us characterize the chemical features at the binding interfaces in different protein complexes.

## Conclusion

Protein–protein interactions underlie many important biological processes [65–67]. The quantitative estimation of how fast these interactions can be formed has broad implications to protein design [68] and drug discovery [69]. The improvement of experimental techniques and the collection of high-throughput experimental data on protein–protein association facilitate the development of computational approaches to model and predict association rates. In this article, by integrating a coarse-grained simulation approach into a neural-network-based classification model, we proposed a biophysical informatics platform to estimate whether the association rate of a protein complex is higher than a predefined threshold or not. This platform has been tested against a large-scale protein complex benchmark selected from the SKEMPI database. The cross-validation results show that, when an optimal threshold was selected, we can reach the best performance with specificity, precision, sensitivity and overall accuracy all higher than 70%. The quality of our cross-validation data has further been testified by the statistical analysis of ROC curve. By looking into the individual cases in which our method failed to classify the protein complexes into their corresponding groups of association rates, we suggest that our model can be improved in the future by implementing the conformational dynamics of proteins into the simulations of their association. Finally, given an independent testing set containing ten additional protein complexes, we can successfully predict the group of their association rates for eight. Taken together, our computational model serves as a useful addition to a collection of existing experimental approaches that measure protein–protein association rates.

## Methods

### The collection of protein complex benchmark set for rate classification from SKEMPI

The experimental data of protein–protein association rates used in this study were derived from the SKEMPI database. It is a comprehensive database that contains not only the absolute values but also the changes of binding constants for wild-type and mutated protein complexes. Similar information was also provided in a previous benchmark study, in which the structures and binding affinities of 179 protein complexes were included [70]. The most updated version, SKEMPI 2.0, includes data of 345 wild-type protein complexes and their 7085 associated mutants [71]. All these data is available online at https://life.bsc.es/pid/skempi2/. The structures of all the wild-type complexes in the database are also available in the protein databank, while the structures of mutants were computationally modeled and can be downloaded from the database. In order to avoid second-ordered error in our simulations, only wild-type protein complexes were considered in this study. Moreover, among all the 345 wild-type protein complexes, only 114 contain the information of association rates. Most of these rate constants were measured using SPR or IASP.

For these 114 entries, we further removed the protein complexes with non-consistent experimental data of association rates that were collected from different studies. The protein complexes with irregular binding interfaces or untypical association pathways were also eliminated from the final benchmark. For instance, the complex formed between transcriptional coactivator CBP/p300 and nuclear receptor p160 (PDB 1KBH) exists as a cooperatively folded helical heterodimer. The association of this type of complexes cannot be simulated by our method. They are thus excluded in the study. Consequently, the number of protein complexes in the benchmark has further been narrowed down from 114 to 102. Coarse-grained Monte-Carlo simulations were carried out for protein complexes of all these remaining entries. However, we only successfully generated simulation trajectories under all different values of distance cutoff for 96 out of 102 protein complexes. The PDB of six entries which failed to complete all the simulation runs are: 1A4Y; 1WQJ; 2B42; 2NY7; 3BT1; and 4K71. Simulations in these systems were aborted under small values of distance cutoff. As a result, a total number of 96 protein complexes were passed into our final prediction model by feeding them into the neural network for association-rate classification. Detailed information about this benchmark set can be found at https://github.com/wulab-github/KonPred.

### A residue-based Monte-Carlo algorithm for simulating association between proteins

For each given protein complex in the benchmark, the process of its association from separated binding partners was modeled by a previously developed kinetic Monte-Carlo simulation method. In specific, a coarse-grained model of protein structures is used in the simulation. Comparing with other previous coarse-grained models in which protein sidechains were grouped into either one [72] or multiple beads [73], each residue of a protein here is represented by the Cα atom plus the representative center of its side-chain which is selected based on the specific properties of the amino acid. The simulation starts from an initial conformation, in which two separated binding partners of a protein complex are placed randomly whereas their corresponding binding interfaces are separated under the range of a given distance cutoff *d*_*c*_ [74]. Specifically, this is calculated as the distance between the centers of mass of all residues within the known interfaces of two binding partners. Moreover, when we generated the initial conformation, the relative orientations between two binding partners were left as random. Following the initial conformation, each protein diffuses randomly within one simulation step. A physics-based scoring function is used to guide the diffusions of proteins during simulations. The scoring function contains a term evaluating the electrostatic interaction which was adopted from the Kim-Hummer model [75, 76], as well as a term estimating the hydrophobic effect between proteins which was taken from a previous study by Kyte and Doolittle [77]. Based on the calculated energy, Metropolis criterion [78] is applied to determine the probability that accepts the corresponding diffusional movements. The simulation trajectory will be terminated if an encounter complex is formed at the end of each simulation step through the corresponding interface. Otherwise, above simulation procedure will be repeated until it reached the maximal time duration.

Practically, this simulation algorithm is performed in parallel under 10 different distance cutoffs from 16 to 25 Å (Fig. 1a). If the distance cutoff is set to be smaller than 16 Å, the separations of the binding interfaces between two interacting proteins will not be far away enough, which leads into stereochemical clashes during the generation of initial configurations for some protein complexes in the benchmark. On the other hand, the association probability drops along with the increase of distance cutoff. As shown in Fig. 3c, the association probabilities for most protein complexes in the benchmark have already reached to 0 when the distance cutoff equals 25 Å. It would be less meaningful if we spend computational resources in simulations with further larger distance cutoff. As a result, simulations in this study were carried out using distance cutoff within the range between 16 and 25 Å. Given a specific value of distance cutoff, 10^3^ trajectories are carried out. Each trajectory consists of 10^3^ steps and each step is 0.01 ns, so that the total simulation time for each trajectory is 10 ns. Moreover, each trajectory starts from a relatively different initial conformation, including different relative orientation between the interfaces of two binding partners. However, their initial distances are all below the given cutoff value in these trajectories.

Encounter complexes can be successfully formed within some of these 10^3^ trajectories, while proteins diffuse away from each other at the end of other trajectories (Fig. 1a). We assume that an encounter complex can be formed when there are at least three native contacts restored in the complex. An intermolecular interaction formed between two residues is considered to be restored if the distance between the representative centers of these two residues is less than 2 Å from the distance observed in the native structure of the protein complex. Based on counting how many encounter complexes formed among all the 10^3^ trajectories, the probability of association under each specific value of distance cutoff can be derived. Finally, the association profile of a protein complex consists of a total dimension of 10 probabilities, which correspond to the calculated association probabilities under the distance cutoffs from 16 to 25 Å. As a result, these profiles for all the protein complexes from the benchmark will be fed into the neural network model as input for association rate classification (Fig. 1a).

The reliability of the method to model protein–protein association has further been validated by systematically adjusting the simulation parameters. Details about the model validation can be found in the Additional file 1.

### Neural-network-based classification of protein–protein association rates

A feedforward back-propagation network was implemented to classify protein–protein association rates. For a specific protein complex, the input neurons of the network are in ten dimensions. As described in the last section, each dimension gives the probability of association that was calculated from the Monte-Carlo simulation under the distance cutoff from 16 to 25 Å. With the given inputs of association profile (Fig. 1b), the output is in one dimension, which informs whether the association rate is higher than a predefined threshold or not (Fig. 1b). The network further contains a single hidden layer with four neurons. A sigmoid activation function was adopted. Weight of each neuron is modified using the back-propagation learning algorithm with a sum of square error function [74]. The magnitude of the error sum in the learning process is monitored in each cycle. The learning is terminated when the network converges.

In order to calibrate the classification performance, the leave-one-out cross-validation strategy was applied to the benchmark set. During the cross-validation, one protein complex was selected from the benchmark for testing, while the remaining 95 entries were considered as the training set. The complexes in the training set were assigned into two classes based on comparing the experimentally determined association rate of each complex with the threshold. A complex belongs to class one if its association rate is lower than the threshold, otherwise it belongs to class two. Both inputs and outputs of training set were fed into the neural network model. After training, the association profile of the selected testing protein complex was used as input for prediction. The predicted outcome was compared with the real association rate. After above procedure is gone through all protein complexes in the benchmark for testing, the overall performance of classification can be attained by calculating the true positive rate (TPR) and false positive rate (FPR) from the summary of each individual complex, as well as the specificity, sensitivity, precision and accuracy of the overall prediction. Detailed evaluation of our cross-validation results is described in the “Results and Discussions”.

The classification program is available for download at: https://github.com/wulab-github/KonPred. This package contains an executable file predicting if the association rate of two binding partners in an input protein complex is higher than a predefined threshold. It also contains the list of 96 protein complexes in the benchmark set and their calculated association profiles used as input for the neural network model. The package offers an instruction and a demonstration example (PDB 7CEI) of how to obtain the prediction with the templates of both input and output files. The program work on a Linux platform and downloading is free for academic users.

## Supplementary Information

**Additional file 1.** The Supporting Information contains the model validation for the Monte-Carlo simulations of protein-protein association; the supporting figures from Figure S1 to Figure S6; and supporting Table S1 and Table S2.

## Data Availability

The data and source code are freely available at https://github.com/wulab-github/KonPred.

## Abbreviation

SKEMPI: Structural database of kinetics and energetics of mutant protein interactions
