# Supplementary material for: Classification of protein–protein association rates based on biophysical informatics
Source: BMC Bioinformatics. 2021 Aug 17;22:408. doi: 10.1186/s12859-021-04323-0 (PMC8371850; doi:10.1186/s12859-021-04323-0)
Supplement: Supplementary file 1 — Additional file 1. The Supporting Information contains the model validation for the Monte-Carlo simulations of protein-protein association; the supporting figures from Figure S1 to Figure S6; and supporting Table S1 and Table S2. [file 12859_2021_4323_MOESM1_ESM.pdf]

Classification of Protein-protein Association Rates Based on Biophysical Informatics

Kalyani Dhusia, and Yinghao Wu

Supporting Information

Model validation for the Monte-Carlo simulations of protein-protein association

In order to evaluate the reliability of our residue-based kinetic Monte-Carlo simulation method, we systematically adjusted different parameters in the simulation. We first tested the effects of different energetic contributions on regulating the protein-protein association. The scoring function used in our Monte-Carlo simulation contains two energy terms. One is the electrostatic interaction and the other is the hydrophobic interaction between two interacting proteins in the system. We have carried out two specific tests. In one test we turned off the contribution of the electrostatic interaction by setting its weight to 0, while in the other test we turned off the contribution of the hydrophobic interaction. We further compared their simulation results with the situation in which both electrostatic and hydrophobic interactions are on. In detail, the protein complexes 2VLN and 2VLR were used as two test systems. The experimental association rate of 2VLN is $1\times10^{8}M^{-1}s^{-1}$. This rate constant is at the upper bound of diffusion-limited region, in which protein-protein associations are dominated by long-range electrostatic interactions. In contrast, the association of 2VLR is much slower, with a rate of $5\times10^{4}M^{-1}s^{-1}$. For both complexes, ten different values of distance cutoff from 16Å to 25Å were test for the system without electrostatic interaction, as well as for the system without hydrophobic interaction. Under each condition, 10^{3} independent simulation trajectories were carried out and the association probability was then calculated. These calculated probabilities are summarized in **Figure S2** as a function of distance cutoff. The systems without hydrophobic interaction are shown by red circles. The systems without electrostatic interaction are shown by blue triangles, while the systems with both electrostatic and hydrophobic interactions are shown by black squares. As shown in **Figure S2a,** the association of 2VLN has been completely diminished when the electrostatic interaction was turned off. On the other hand, there are few impacts on its association when the hydrophobic interaction was turned off. This result indicates that the association of complex 2VLN is dominated by its electrostatic interaction. Different from 2VLN, the elimination of either electrostatic or hydrophobic interaction did not fully block the association of complex 2VLR, as shown in **Figure S2b**. This result indicates that its association is regulated by the combination of both effects. Our simulations therefore show the evidence that protein-protein association is controlled by both electrostatic and hydrophobic interactions, and in a case-dependent manner. We also suggest that while the domination of long-range electrostatic interaction leads into fast association, short-range hydrophobic interaction plays a more important role in slow association.

In the next step, we tested the calculation of association probability by changing different numbers of simulation trajectories. In our original study, 10^{3} independent simulation trajectories were carried out under different values of distance cutoff. Larger numbers of trajectories were further tried. In detail, the protein complex 2VLN was used as a test model. For each distance cutoff from 16Å to 25Å, three scenarios were generated. In the first scenario, 10^{3} independent simulation trajectories were performed; in the second scenario, 2×10^{3} independent simulation trajectories were performed; and in the third scenario, 5×10^{3} independent simulation trajectories were performed. Under each value of distance cutoff and each scenario, the numbers of trajectories in which encounter complexes were successfully formed have been recorded. The results are summarized in **Figure S3a**. The black squares in the figure correspond to the scenario with 10^{3} trajectories, while the red circles and blue triangles correspond to the scenarios with 2×10^{3} and 5×10^{3}, respectively. These numbers of successful association have further been converted to the association probabilities by dividing with the total numbers of trajectories. The derived association probabilities for all three scenarios are shown in **Figure S3b**. The figure

indicates that the profiles of association probability from different scenarios are very close to each other. This result suggests that the calculations of association probability were converged. Further increase of simulation trajectories did not affect the outcome. As a result, 10^3 simulation trajectories are sufficient to obtain statistically meaningful results of association probabilities.

We further calibrated the acceptance rate in our Monte-Carlo simulations. The calibration was carried out by adjusting the simulation temperature which is embedded in the Metropolis Criteria of the algorithm. In detail, the protein complex 2VLN was used as a test model. Five different values of temperature were tried. They are $0.2T_0$, $0.5T_0$, T_0, $2T_0$, and $5T_0$, in which T_0 is the room temperature used in our original model. For each temperature, distance cutoff was variated from 16Å to 25Å, while 10^3 independent simulation trajectories were generated under each distance cutoff value. We calculated the acceptance rate of Monte-Carlo movements from the 1000 simulation steps within each of these trajectories. The average values of these calculated acceptance rates were plotted together with their standard deviation in **Figure S4a** as a function of simulation temperature. Not surprisingly, the figure shows that the acceptance rate becomes higher when the temperature increases, while the standard deviation of their distributions becomes smaller. Under the temperature of T_0, the overall acceptance rate equals 0.87. We also plotted the association rates as a function of distance cutoff under different simulation temperatures in **Figure S4b**. The association profiles in the figure shows that the overall probabilities drop as the temperature increases. Under low temperature, the energetically unfavored moves are more likely to be rejected. As a result, the Monte-Carlo acceptance rate becomes low but the proteins are more likely to find their native-like binding conformation which leads to the increase of association probabilities. In contrast, under high temperature, the energetically unfavored moves are more likely to be accepted. As a result, the Monte-Carlo acceptance rate becomes high but the proteins are less likely to find their native-like binding conformation which leads to the decrease of association probabilities.

Finally, we changed the lengths of each time step in the simulations. In our original model, each trajectory consists of 10^3 steps and each time step is 0.01 ns, so that the total simulation time duration for each trajectory is 10 ns. In addition to this setting, two scenarios were further tested. In the first scenario, we changed the length of each simulation time step into 0.1ns. In order to keep the total time duration of each trajectory unchanged, the number of simulation steps was reduced from 1000 to 100. In the other scenario, we changed the length of each simulation time step into 0.001ns. In order to keep the total time duration of each trajectory unchanged, the number of simulation steps was increased from 1000 to 10000. All three scenarios, including the original setting, were applied to the test model 2VLN. In detail, within each scenario, 10^3 independent simulation trajectories were performed for each distance cutoff from 16Å to 25Å and the association probabilities were further calculated. The derived association probabilities for all three scenarios are summarized in **Figure S5**. The simulation results from the system which time step equals 0.001ns are plotted by red circles. The simulation results from the system which time step equals 0.01ns are plotted by black squares. The simulation results from the system which time step equals 0.1ns are plotted by blue triangles. All three scenarios show that the association probabilities drop as the distance cutoff increases. However, with larger time step (0.1ns), much lower association probabilities were derived, indicating that the simulation with only 100 steps for each trajectory is not sufficient. On the other hand, the association profiles derived from the other two time-steps are very close, indicating that the setting of the time step in our original model is sufficient for conformational sampling.

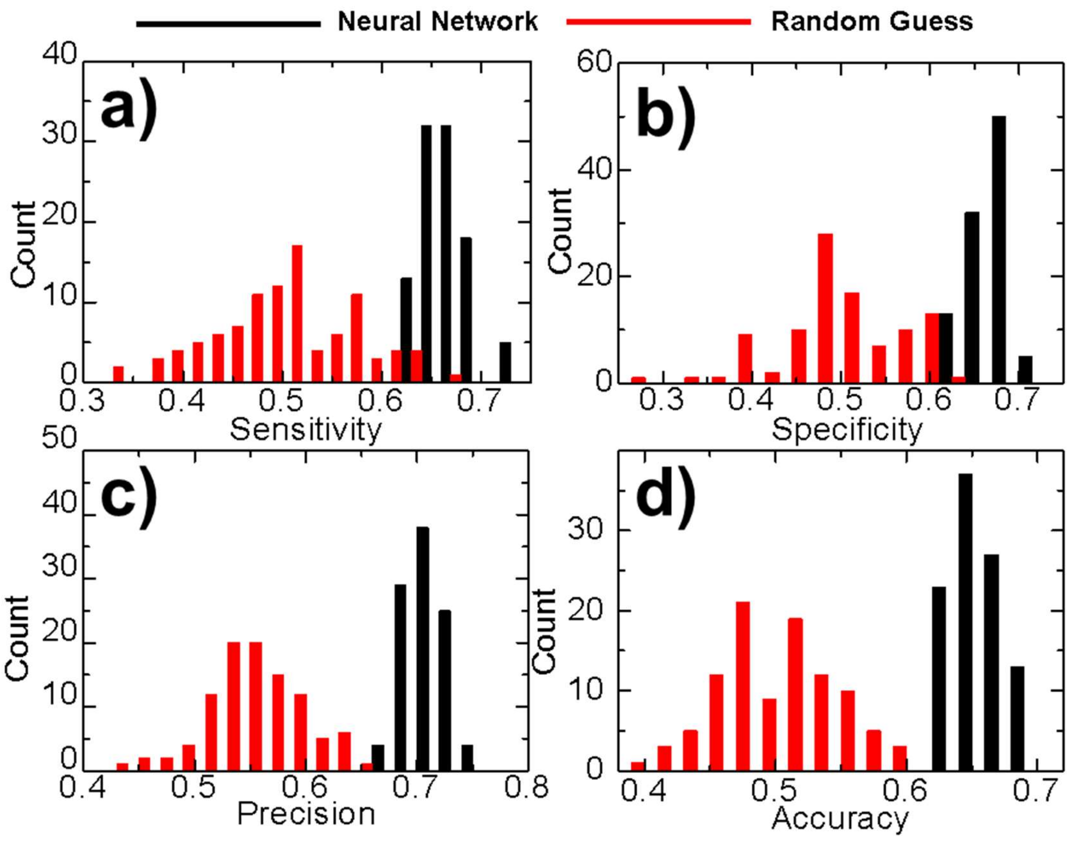

Figure S1: The comparison of the prediction results from our neural-network-based classification method (black) with the prediction results from random estimation (red) for sensitivity **(a)**, specificity **(b)**, precision **(c)** and accuracy **(d)**, respectively.

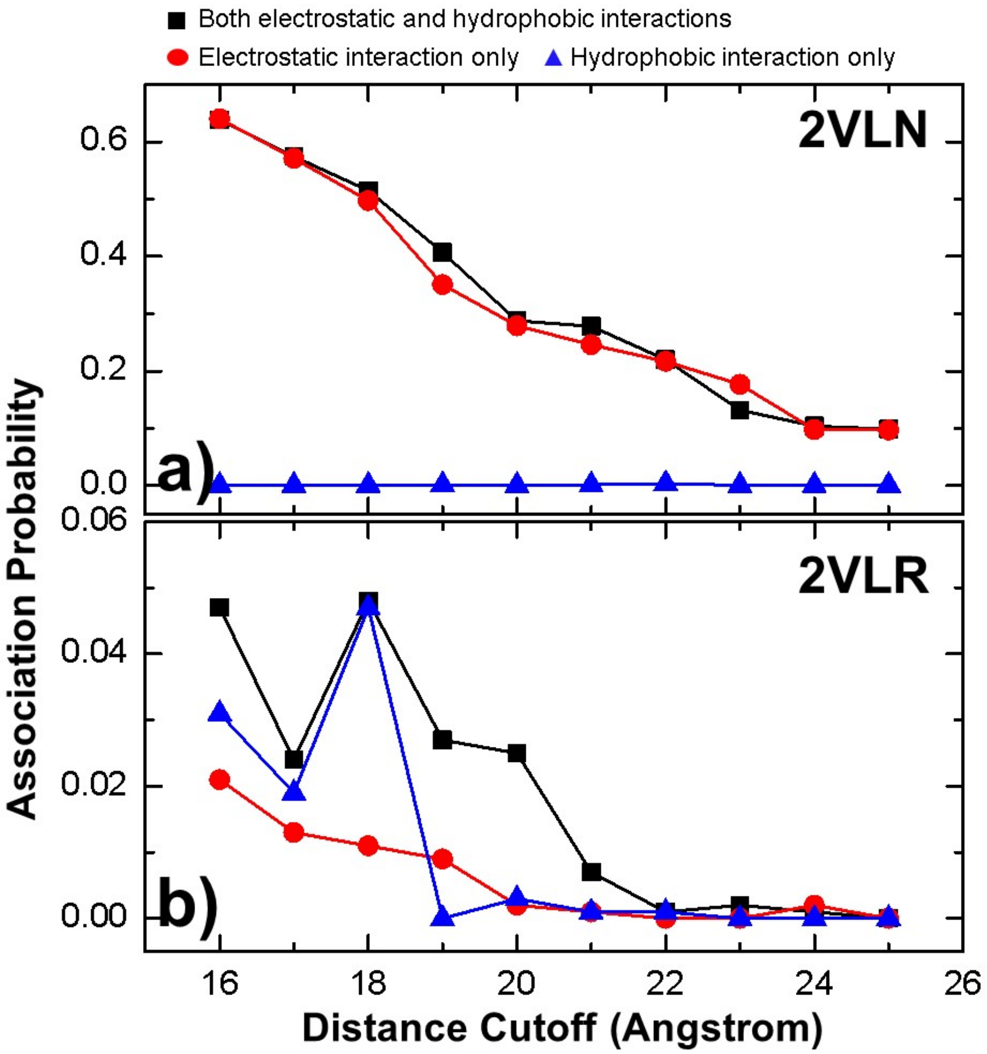

Figure S2: We tested the effects of different energetic contributions on regulating the protein-protein association

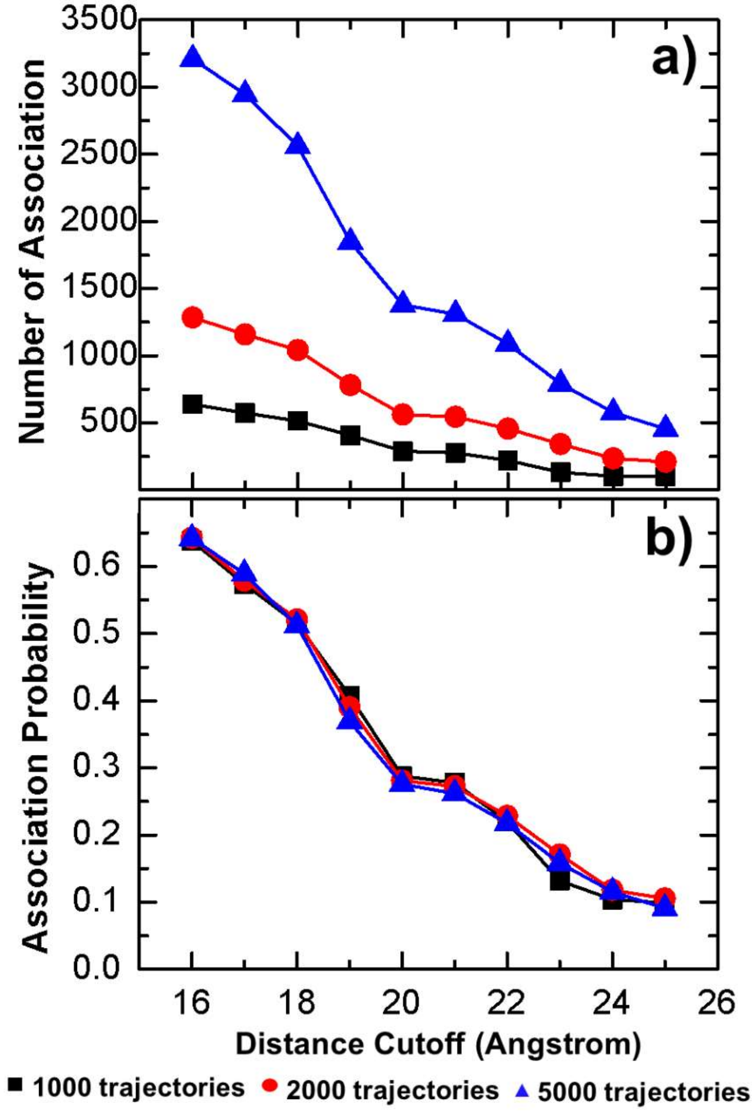

Figure S3: We tested the calculation of association probability by changing different numbers of simulation trajectories.

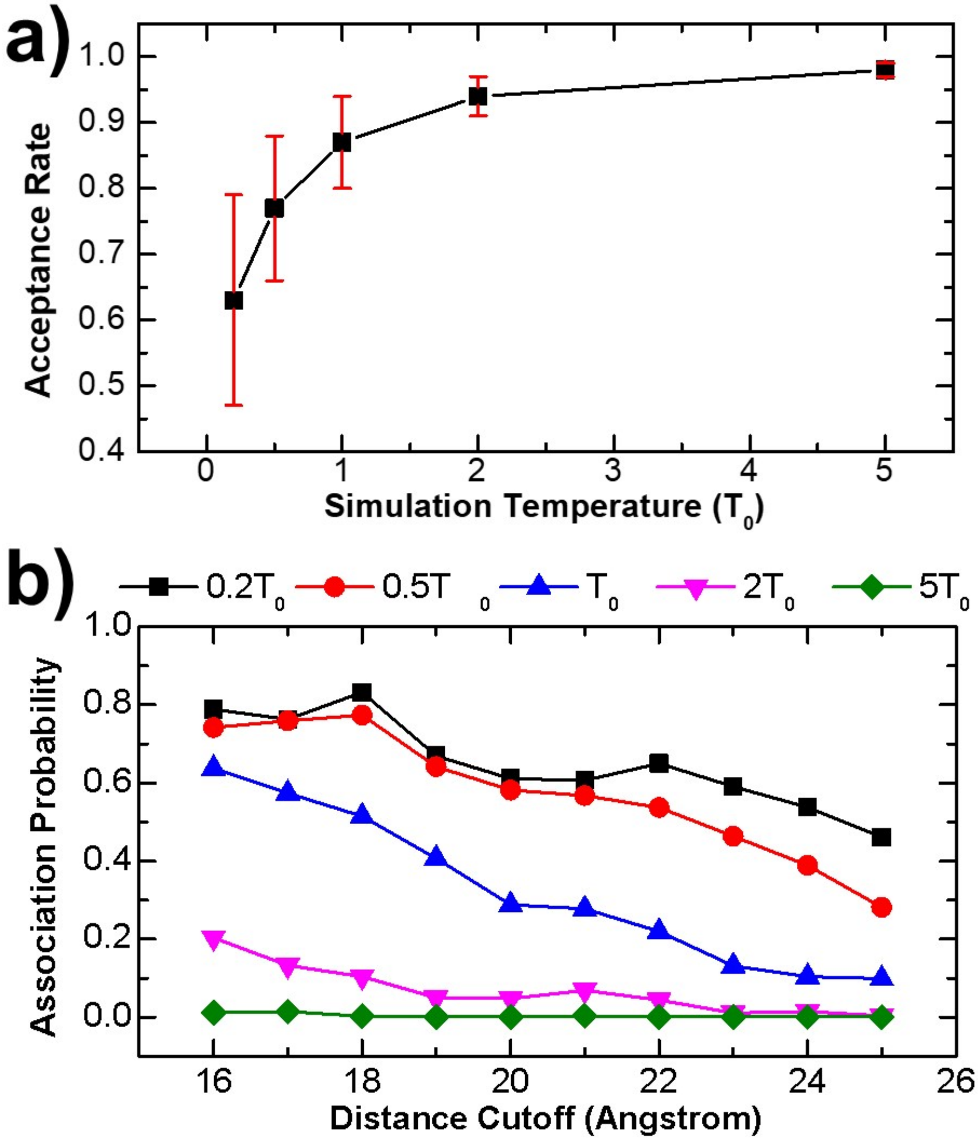

Figure S4: We calibrated the acceptance rate in our Monte-Carlo simulations by adjusting the simulation temperature.

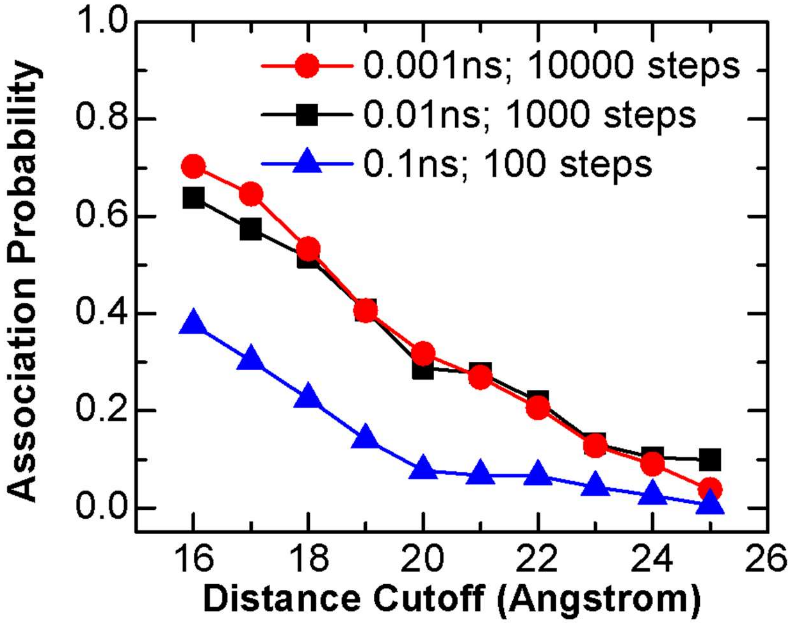

Figure S5: We tested the calculation of association probability by changing the lengths of each time step in the simulations.

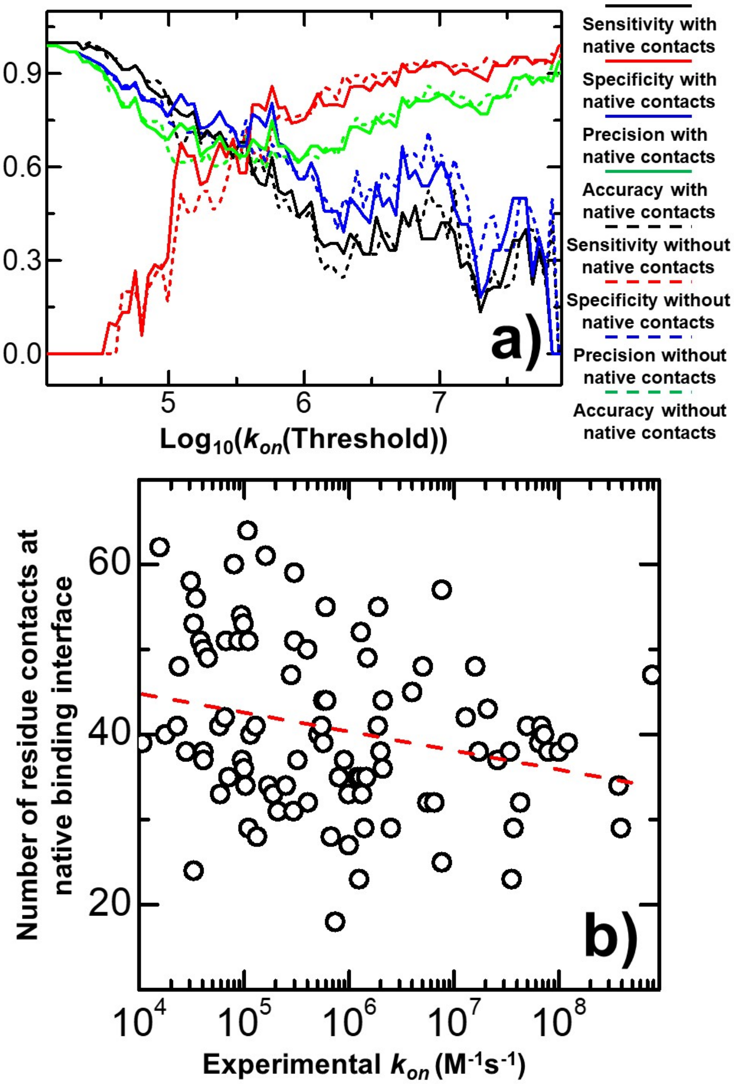

Figure S6: The number of intermolecular contacts between residues at native binding interfaces was used as an additional dimension of inputs to the neural-network classification. The classification outcomes were compared with the original results in **(a)**. We further plotted the correlation between the native contacts and the experimentally measured association rates for all 96 protein complexes in the benchmark **(b)**.

		# of samples	Mean	S. D.	Minimum	Median	Maximum
Neural Network	Sensitivity	100	0.65	0.030	0.57	0.66	0.73
	Specificity	100	0.67	0.025	0.63	0.67	0.72
	Precision	100	0.71	0.019	0.67	0.71	0.75
	Accuracy	100	0.66	0.020	0.63	0.65	0.70
Random Guess	Sensitivity	100	0.49	0.067	0.32	0.50	0.66
	Specificity	100	0.49	0.067	0.28	0.48	0.62
	Precision	100	0.55	0.042	0.44	0.54	0.64
	Accuracy	100	0.49	0.043	0.39	0.48	0.59

Table S1: The comparison of statistical distributions between results from neural network and results from random estimation.

PDB	Partner 1	Partner 2	k_{on}(EXP) [$M^{-1}s^{-1}$]	Log(k_{on})	Category	Pred_P/N	T/F	P_{ass} (16Å)	P_{ass} (17Å)	P_{ass} (18Å)	P_{ass} (19Å)	P_{ass} (20Å)	P_{ass} (21Å)	P_{ass} (22Å)	P_{ass} (23Å)	P_{ass} (24Å)	P_{ass} (25Å)
1A22	A	B	300000	5.47712	-1	0.3658	0	0.031	0.215	0.049	0.009	0.026	0.019	0.004	0.007	0.002	0
1AO7	ABC	DE	23000	4.36173	-1	-0.5371	1	0.032	0.014	0.004	0.012	0.006	0.003	0	1E-3	0.002	0.003
1B2S	A	D	4.3E7	7.63347	1	0.04098	1	0.172	0.139	0.096	0.118	0.046	0.025	0.044	0.037	0.007	1E-3
1B2U	A	D	3.7E7	7.5682	1	0.10978	1	0.232	0.219	0.143	0.148	0.103	0.057	0.038	0.026	0.015	0.002
1B3S	A	D	3.9E8	8.59106	1	0.39882	1	0.243	0.258	0.218	0.16	0.046	0.051	0.042	0.066	0.051	0.002
1B41	A	B	5.05E6	6.70329	1	0.74335	1	0.467	0.472	0.326	0.288	0.226	0.167	0.096	0.11	0.09	0.066
1BD2	ABC	DE	96000	4.98227	-1	0.56335	0	0.168	0.136	0.147	0.108	0.043	0.048	0.045	0.011	0.004	0.009
1BJ1	HL	VW	41000	4.61278	-1	-0.45116	1	0.108	0.063	0.058	0.035	0.027	0.02	0.016	0.013	0.017	0.005
1BRS	A	D	3.7E8	8.5682	1	0.8106	1	0.412	0.332	0.341	0.263	0.223	0.13	0.115	0.099	0.068	0.038
1CBW	FGH	I	170000	5.23045	-1	-0.7776	1	0.089	0.011	0.037	0.006	0.011	0	1E-3	0.006	0.004	0
1CZ8	HL	VW	41000	4.61278	-1	-0.6379	1	0.134	0.1	0.06	0.03	0.05	0.032	0.021	0.009	0.004	0.016
1EMV	A	B	7.24E7	7.85974	1	0.95429	1	0.772	0.678	0.612	0.496	0.359	0.307	0.211	0.182	0.183	0.142
1FC2	C	D	190000	5.27875	-1	-0.33581	1	0.076	0.083	0.069	0.043	0.02	0.025	0.009	0.004	0.006	1E-3
1FR2	A	B	2.6E7	7.41497	1	0.18363	1	0.645	0.396	0.348	0.359	0.182	0.169	0.151	0.13	0.126	0.105
1FSS	A	B	6.8E7	7.83251	1	0.96102	1	0.84	0.772	0.654	0.51	0.445	0.309	0.217	0.113	0.103	0.152
1GL0	E	I	103000	5.01284	-1	-0.58544	1	0.078	0.045	0.056	0.023	0.002	0	1E-3	0	1E-3	0.002
1GL1	A	I	800000	5.90309	1	-0.98593	0	0.169	0.077	0.034	0.003	0.007	1E-3	0.004	1E-3	0	0
1IAR	A	B	1.3E7	7.11394	1	0.89716	1	0.864	0.825	0.766	0.688	0.606	0.549	0.452	0.429	0.405	0.329
1JRH	LH	I	609000	5.78462	1	0.73888	1	0.345	0.305	0.286	0.235	0.134	0.144	0.13	0.052	0.057	0.053
1JTD	A	B	7.7E6	6.88649	1	-0.86754	0	0.215	0.126	0.134	0.075	0.122	0.086	0.036	0.02	0.024	0.008
1JTG	A	B	108000	5.03342	-1	0.96768	0	0.337	0.121	0.268	0.219	0.18	0.123	0.097	0.056	0.024	0.047
1KTZ	A	B	740000	5.86923	1	0.56946	1	0.454	0.441	0.351	0.239	0.208	0.133	0.102	0.079	0.089	0.061
1LFD	A	B	7.7E6	6.88649	1	0.51402	1	0.343	0.317	0.263	0.216	0.135	0.131	0.14	0.11	0.1	0.096
1LP9	ABC	EF	31000	4.49136	-1	0.34569	0	0.338	0.23	0.166	0.185	0.146	0.112	0.103	0.097	0.077	0.061
1MAH	A	F	7.8E8	8.89209	1	0.52356	1	0.36	0.284	0.301	0.248	0.072	0.154	0.165	0.079	0.118	0.062
1MHP	HL	A	250000	5.39794	-1	0.10294	0	0.046	0.003	0.057	0.023	0.018	0.012	0.007	0.003	0.041	0.029
1MI5	ABC	DE	34800	4.54158	-1	-0.24671	1	0.039	0.013	0.005	0.022	0.005	0.012	0.007	0.022	0.016	0.038
1MLC	AB	E	2.5E6	6.39794	1	0.12056	1	0.114	0.069	0.087	0.062	0.05	0.025	0.023	0.042	0.024	0.043
1MQ8	A	B	133000	5.12385	-1	-0.08653	1	0.052	0.067	0.05	0.028	0.022	0.021	0.015	0.036	0.022	0.004
1NMB	N	LH	161000	5.20683	-1	0.99254	0	0.814	0.649	0.584	0.498	0.457	0.384	0.299	0.246	0.207	0.195
1OGA	ABC	DE	28000	4.44716	-1	-0.75268	1	0.123	0.05	0.031	0.019	0.022	0.034	0.02	0.013	0.009	1E-3
1QSE	ABC	DE	58000	4.76343	-1	-0.44963	1	0.129	0.135	0.085	0.022	0.072	0.057	0.033	0.034	0.048	0.007
1REW	AB	C	600000	5.77815	1	-0.99218	0	0.078	0.436	0.161	0.036	0.118	0.024	0.128	0.107	0.075	0.086
1TM1	E	I	5.6E6	6.74819	1	-0.57723	0	0.161	0.099	0.121	0.056	0.024	0.002	0.016	0.019	0.029	0.014
1X1X	A	D	897000	5.95279	1	0.77579	1	0.325	0.363	0.287	0.224	0.172	0.146	0.093	0.042	0.033	0.075
1YY9	CD	A	1.46E6	6.16435	1	0.1105	1	0.013	0.031	0.019	0.023	0.01	0.009	0.01	0.005	0.002	1E-3
2AJF	A	E	71200	4.85248	-1	0.04138	0	0.031	0.043	0.011	0.025	0.048	0.038	0	0.008	0.002	0.006
2AK4	ABC	DE	65600	4.8169	-1	-0.07187	1	0.007	0.009	0.004	0.012	0.002	0.021	0.006	0.011	0.004	0.002
2BNR	ABC	DE	24000	4.38021	-1	0.31138	0	0.168	0.118	0.144	0.066	0.075	0.064	0.057	0.039	0.046	0.034
2C5D	A	C	2.1E7	7.32222	1	0.98728	1	0.906	0.824	0.757	0.685	0.622	0.554	0.43	0.325	0.26	0.278
2DSQ	I	G	671000	5.82672	1	0.60838	1	0.118	0.099	0.127	0.091	0.063	0.082	0.047	0.055	0.007	0.005
2FTL	E	I	990000	5.99564	1	-0.37814	0	1E-3	0.004	0	0	0	0	1E-3	0	0	0
2GOX	A	B	404000	5.60638	1	0.95158	1	0.64	0.578	0.447	0.415	0.35	0.3	0.233	0.221	0.214	0.135
2GYK	A	B	1.73E7	7.23805	1	0.31006	1	0.388	0.208	0.256	0.17	0.127	0.069	0.074	0.106	0.03	0.006
2I26	N	L	210000	5.32222	-1	-0.04711	1	0.084	0.048	0.049	0.064	0.007	0.027	0.004	0.041	0.046	1E-3
2JCC	ABC	EF	41000	4.61278	-1	0.73539	0	0.308	0.233	0.218	0.178	0.139	0.101	0.103	0.092	0.096	0.057
2P5E	ABC	DE	570000	5.75587	1	-0.84939	0	0.227	0.175	0.086	0.125	0.047	0.031	0.02	0.005	0.03	0.019
2PYE	ABC	DE	17800	4.25042	-1	-0.44272	1	0.27	0.194	0.135	0.123	0.083	0.04	0.045	0.04	0.022	0.015
2QJ9	AB	C	400000	5.60206	1	0.8262	1	0.049	1E-3	0.125	0.038	0.099	0.034	0.032	0	0	0.009
2QJA	AB	C	300000	5.47712	-1	0.99977	0	0	0.196	0.253	0.145	0.1	0.034	0.078	0.007	0.025	0.003
2QJB	AB	C	280000	5.44716	-1	-0.99582	1	0.383	0.32	0.115	0.123	0.09	0.111	0.074	0.034	0.065	0.011
2SIC	E	I	6.5E6	6.81291	1	-0.36641	0	0	1E-3	1E-3	0	0	0	0	0	0.012	1E-3
2UWE	ABC	EF	33000	4.51851	-1	0.78831	0	0.438	0.265	0.229	0.223	0.172	0.102	0.09	0.125	0.091	0.078
2VIR	AB	C	110000	5.04139	-1	-0.28832	1	0.179	0.178	0.106	0.109	0.026	0.055	0.066	0.037	0.019	0.006
2VLN	A	B	1E8	8	1	0.91876	1	0.634	0.597	0.507	0.386	0.291	0.263	0.217	0.142	0.116	0.101
2VLO	A	B	3.42E7	7.53403	1	0.67214	1	0.466	0.368	0.329	0.244	0.211	0.185	0.154	0.057	0.08	0.047
2VLP	A	B	6.52E7	7.81425	1	0.90968	1	0.332	0.459	0.356	0.226	0.245	0.266	0.214	0.141	0.098	0.087
2VLQ	A	B	7.91E7	7.89818	1	0.84134	1	0.681	0.483	0.446	0.301	0.294	0.276	0.236	0.198	0.182	0.133
2VLR	ABC	DE	59000	4.77085	-1	0.14644	0	0.047	0.024	0.048	0.027	0.025	0.007	1E-3	0.002	0.008	1E-3
2WPT	A	B	5E7	7.69897	1	0.95844	1	0.63	0.543	0.493	0.429	0.341	0.325	0.248	0.161	0.117	0.07
3BDY	HL	V	33000	4.51851	-1	-0.04486	1	0.006	1E-3	0.008	0.005	0	1E-3	0	0	1E-3	0
3BE1	HL	A	100000	5	-1	-0.20071	1	1E-3	0	0	0	0	0	0	0	0	0
3BK3	A	C	1.4E6	6.14613	1	-0.08962	0	0	0	0.002	0	0.002	0	1E-3	0.009	0.004	0
3BP8	A	C	995000	5.99782	1	-0.24893	0	0.005	0.004	0.003	1E-3	0	0	0	0.002	1E-3	1E-3
3D5R	A	C	1.2E6	6.07918	1	0.97616	1	0.802	0.725	0.653	0.571	0.455	0.355	0.294	0.245	0.236	0.198
3D5S	A	C	1.33E6	6.12385	1	0.93169	1	0.781	0.75	0.619	0.528	0.461	0.359	0.295	0.261	0.223	0.2
3HFM	HL	Y	1.88E6	6.27416	1	0.94607	1	0.197	0.197	0.199	0.21	0.195	0.159	0.157	0.15	0.147	0.13
3L5X	A	HL	1.25E6	6.09691	1	0.83676	1	0.113	0.1	0.146	0.119	0.106	0.078	0.064	0.043	0.044	0.038
3LB6	A	C	1.22E8	8.08636	1	0.44415	1	0.009	0.002	0.033	0.021	0.01	0.01	0	1E-3	0	0
3MZG	A	B	94000	4.97313	-1	-0.74082	1	0.193	0.13	0.102	0.047	0.01	0.05	0.011	0.006	0.002	0.002
3MZW	A	B	4E6	6.60206	1	0.12006	1	0.368	0.288	0.232	0.18	0.19	0.125	0.092	0.074	0.076	0.061
3N06	A	B	110000	5.04139	-1	-0.79723	1	0.279	0.179	0.134	0.067	0.059	0.019	0.008	0.008	0.007	0.002
3N0P	A	B	99000	4.99564	-1	-0.97482	1	0.247	0.154	0.117	0.057	0.03	0.06	0.005	0.002	0.005	0.005
3N85	A	LH	1.3E6	6.11394	1	-0.18331	0	0.007	0	0.002	0.003	0	0	0	0	0	1E-3
3NCB	A	B	88000	4.94448	-1	-0.98797	1	0.348	0.189	0.123	0.106	0.039	0.013	1E-3	0.002	0.004	0.006
3NCC	A	B	67000	4.82607	-1	-0.9702	1	0.374	0.261	0.155	0.119	0.083	0.039	0.032	0.005	0.006	0.002
3NGB	HL	G	38300	4.5832	-1	-0.68977	1	0.311	0.213	0.127	0.115	0.11	0.106	0.058	0.007	0.018	0.012
3QHY	A	B	1.9E6	6.27875	1	0.94648	1	0.069	0.153	0.145	0.116	0.065	0.027	0.016	0.03	0.052	0.027
3S9D	A	B	2.1E6	6.32222	1	-0.35401	0	0.102	0.135	0.114	0.073	0.01	0.036	0.016	0.022	0.03	0.026
3U82	A	B	570000	5.75587	1	-0.02965	0	0.023	0.029	0.022	0.02	0.009	0.003	0	0.003	0	1E-3
3W2D	A	HL	10700	4.02938	-1	-0.53542	1	0.027	0.009	0.006	1E-3	1E-3	0	0.002	1E-3	0.007	0
4CVW	A	C	1.5E6	6.17609	1	-0.9563	0	0.612	0.199	0.251	0.244	0.23	0.191	0.102	0.192	0.144	0.069
4FTV	ABC	DE	45000	4.65321	-1	-0.59234	1	0.065	0.011	0.006	0.005	0.013	0.005	1E-3	0	0	0
4G0N	A	B	3.55E7	7.55023	1	0.98253	1	0.881	0.844	0.78	0.719	0.631	0.561	0.47	0.449	0.386	0.345
4HFK	A	B	129000	5.11059	-1	-0.74212	1	0.622	0.481	0.292	0.242	0.19	0.13	0.106	0.051	0.024	0.008
4HRN	A	D	322000	5.50786	-1	-0.89054	1	0.114	0.104	0.069	0.012	0.003	0.01	0.015	0.027	0.021	0.007
4JPK	HL	A	15700	4.1959	-1	-0.99439	1	0.154	0.111	0.033	0.006	1E-3	0.013	0.004	1E-3	0.002	0.002
4MNQ	ABC	DE	80000	4.90309	-1	0.30964	0	0.241	0.254	0.185	0.121	0.107	0.054	0.042	0.021	0.026	0.041
4MYW	A	B	545000	5.7364	1	-0.26596	0	0.005	0.038	0.016	0.004	0.005	0.011	1E-3	0	1E-3	1E-3
4RA0	A	C	1.6E7	7.20412	1	0.93141	1	0.752	0.632	0.517	0.469	0.314	0.309	0.222	0.132	0.11	0.08
4U6H	AB	E	512500	5.70969	1	0.87445	1	0.483	0.439	0.365	0.31	0.206	0.123	0.092	0.072	0.067	0.051
4UYP	A	D	2.1E6	6.32222	1	0.11543	1	0.082	0.076	0.057	0.085	0.019	0.018	0.067	0.047	0.049	0.035
4UYQ	A	B	2E6	6.30103	1	-0.81155	0	0.104	0.052	0.05	0.046	0.03	0.035	0.015	0.003	0.006	0.009
5C6T	HL	A	295000	5.46982	-1	-0.29467	1	0.021	0.009	0.005	0.022	0.006	0.014	0.003	0.002	0.012	1E-3
5F4E	A	B	115000	5.0607	-1	-0.4593	1	0.111	0.064	0.022	0.058	0.034	0.04	0.034	0.011	0.042	0.01
5XCO	A	B	1.3E6	6.11394	1	-0.32917	0	0.133	0.144	0.134	0.085	0.121	0.136	0.055	0.015	0.01	0.056

Table S2: Detailed information, simulation and prediction results of all protein complexes in the benchmark

The first column of the table is the PDB id of protein complexes in the benchmark.

The second and third column of the table are the chain id of two interacting partners in the protein complexes.

The fourth column of the table, k_{on}(EXP), is the experimentally measured values of association rates for each protein complex in the unit of $M^{-1}s^{-1}$.

The fifth column of the table, log(k_{on}), is the logarithm value of experimental association rates for each protein complex with base 10.

The sixth column of the table gives the assigned class for each protein complex based on the optimal threshold. The value -1 stands for the class which association rates are lower than the threshold and 1 stands for the other class which association rates are higher than the threshold.

The seventh column of the table is the predicted outputs from the neural network. The negative values mean that the protein complexes were predicted as the class which association rates are lower than the threshold, and the positive values mean that the protein complexes were predicted as the class which association rates are higher than the threshold.

The eighth column of the table indicates whether the prediction is true or false by comparing with the experimental value, with 0 means false and 1 means true.

The numbers shown from the nineth to the eighteenth columns of the table are the probabilities of association calculated from the kinetic Monte-Carlo simulations with the distance cutoff values from 16Å to 25Å.
